# MangoLeafBD: A comprehensive image dataset to classify diseased and healthy mango leaves

**DOI:** 10.1016/j.dib.2023.108941

**Published:** 2023-01-30

**Authors:** Sarder Iftekhar Ahmed, Muhammad Ibrahim, Md. Nadim, Md. Mizanur Rahman, Maria Mehjabin Shejunti, Taskeed Jabid, Md. Sawkat Ali

**Affiliations:** a Department of Computer Science and Engineering, East West University, Dhaka, Bangladesh; b Department of Computer Science and Engineering, University of Dhaka, Dhaka, Bangladesh

**Keywords:** Image classification, Plant disease detection, Machine learning, Precision agriculture, Image dataset

## Abstract

Agriculture is one of the few remaining sectors that is yet to receive proper attention from the machine learning community. The importance of datasets in the machine learning discipline cannot be overemphasized. The lack of standard and publicly available datasets related to agriculture impedes practitioners of this discipline to harness the full benefit of these powerful computational predictive tools and techniques. To improve this scenario, we develop, to the best of our knowledge, the first-ever standard, ready-to-use, and publicly available dataset of mango leaves. The images are collected from four mango orchards of Bangladesh, one of the top mango-growing countries of the world. The dataset contains 4000 images of about 1800 distinct leaves covering seven diseases. Although the dataset is developed using mango leaves of Bangladesh only, since we deal with diseases that are common across many countries, this dataset is likely to be applicable to identify mango diseases in other countries as well, thereby boosting mango yield. This dataset is expected to draw wide attention from machine learning researchers and practitioners in the field of automated agriculture.

**Dataset Specifications**

**Table:** 

Subject	Precision agriculture
Specific subject area	Mango leaf image classification using machine learning.
Type of data	Digital images of size 240 × 320 pixels having RGB color in PNG format.
How the data were acquired	We consider seven mango leaf diseases that affect the mango trees frequently. To collect data from mango gardens of different parts of the country, four mango gardens of Bangladesh were selected based on their size and variety of trees. The leaf images were taken a few days before the winter of 2021. The trees affected by the disease were located – oftentimes with the help of agriculture experts. After collecting the leaves, the images of all the leaves were captured individually using a mobile phone camera with a white background. A total of around 1800 images were recorded.
Data format	Analyzed and filtered.
Description of data collection	We collect the disease-affected (considering seven diseases) leaves and also some healthy leaves directly from the mango trees. Then, the images of these leaves are captured using mobile phone camera. The images are then resized, and some poor quality images are dropped out. Zooming and rotation operations are then performed on these images, thereby giving us a dataset of 4000 images where each of the eight categories has 500 images.
Data source location	The following four mango orchards of Bangladesh are used for data collection: 1. Sher-e-Bangla Agricultural University mango garden in Dhaka (latitude: 23.77157, longitude: 90.37507), 2. Jahangir Nagar University garden in Savar (latitude: 23.87988, longitude: 90.27252), 3. Udaypur village mango garden in Rajbari district (latitude: 23.65966, longitude: 89.68117), and 4. Itakhola village mango garden in Nilphamari district (latitude: 25.98122, longitude: 88.88182).
Data accessibility	**Repository name:** Mendeley data [20]. **Data identification number**:10.17632/hxsnvwty3r.1 **Direct URL to data:**https://data.mendeley.com/datasets/hxsnvwty3r/1

## Value of the Data

The contributions of this research are summarized below:

• We develop the first-ever dataset of mango leaf images of Bangladesh, one of the top mango-growing countries of the world. All the 1800 images are manually captured by the camera from various mango orchards and are then labeled by human experts. Further, after zooming and rotating some images, the size of the dataset reaches 4000.
• We cover a large number of major diseases (seven in particular) that attack the mango trees. The mango trees of Bangladesh are affected mostly by these diseases.
• We apply various data validation techniques that transform the raw dataset into a processed one. The techniques include: noise cleaning, ground-truth labeling by humans, image resizing, zooming, and rotating.
• The dataset is released for public use and is readily available for downloading so researchers can fit the data directly into machine learning systems without any further validation/preprocessing.
• Although the dataset contains images of mango leaves of Bangladesh only, considering its large size, this can easily be used in a transfer learning setting [18] to predict mango diseases of other countries as well.

## 1 Objective

A very promising area of research and innovation is the application of machine learning models for detecting plant diseases from leaf images. So the researchers in this field need readily available representative datasets to develop effective machine learning systems. However, this type of real-life dataset for plant disease detection is not plentiful. From our survey of existing literature, it is evident that there is no standard dataset, in terms of size, noise, class distribution etc., of images of mango leaves. Hence, we think that it is imperative to develop such a dataset and release it to foster research in machine learning-based plant disease detection. Top machine learning scientists and practitioners often opine that the benefit of this discipline is not yet fully harnessed for social goods such as healthcare and agriculture. So our venture is to prepare a standard agricultural dataset that will leap forward, however small, the endeavour of sharing the benefit of machine learning for mass people.

## 2 Data Description

**Fig. 1:**
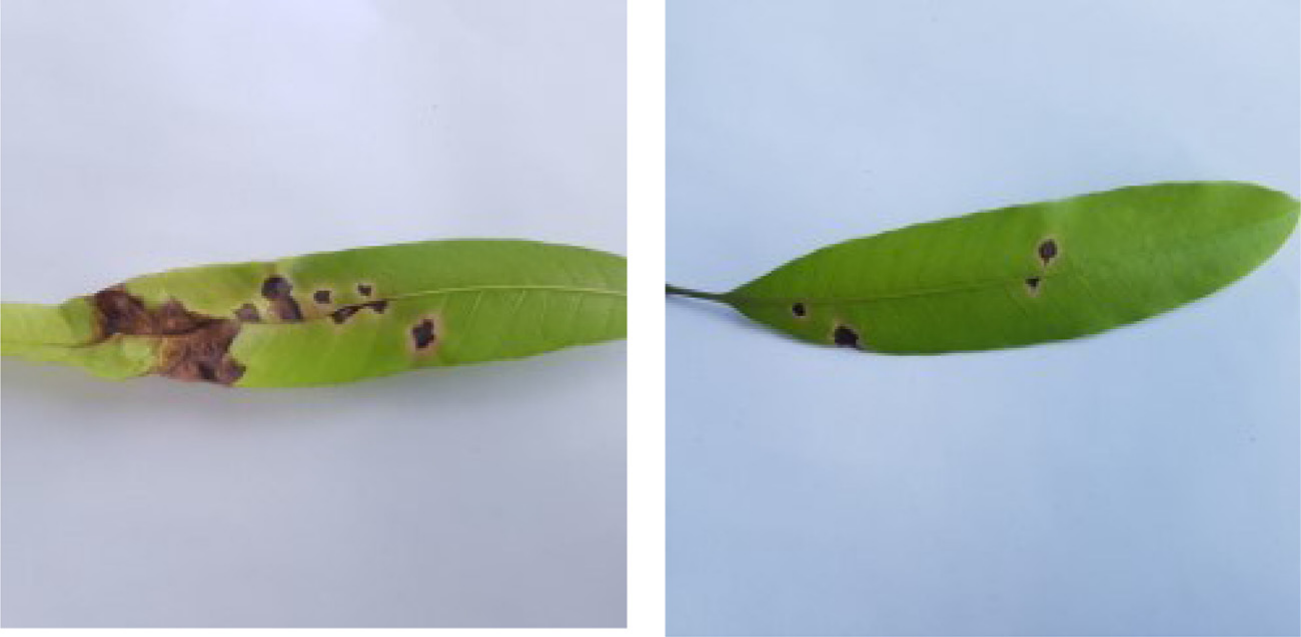
Anthracnose.

**Fig. 2:**
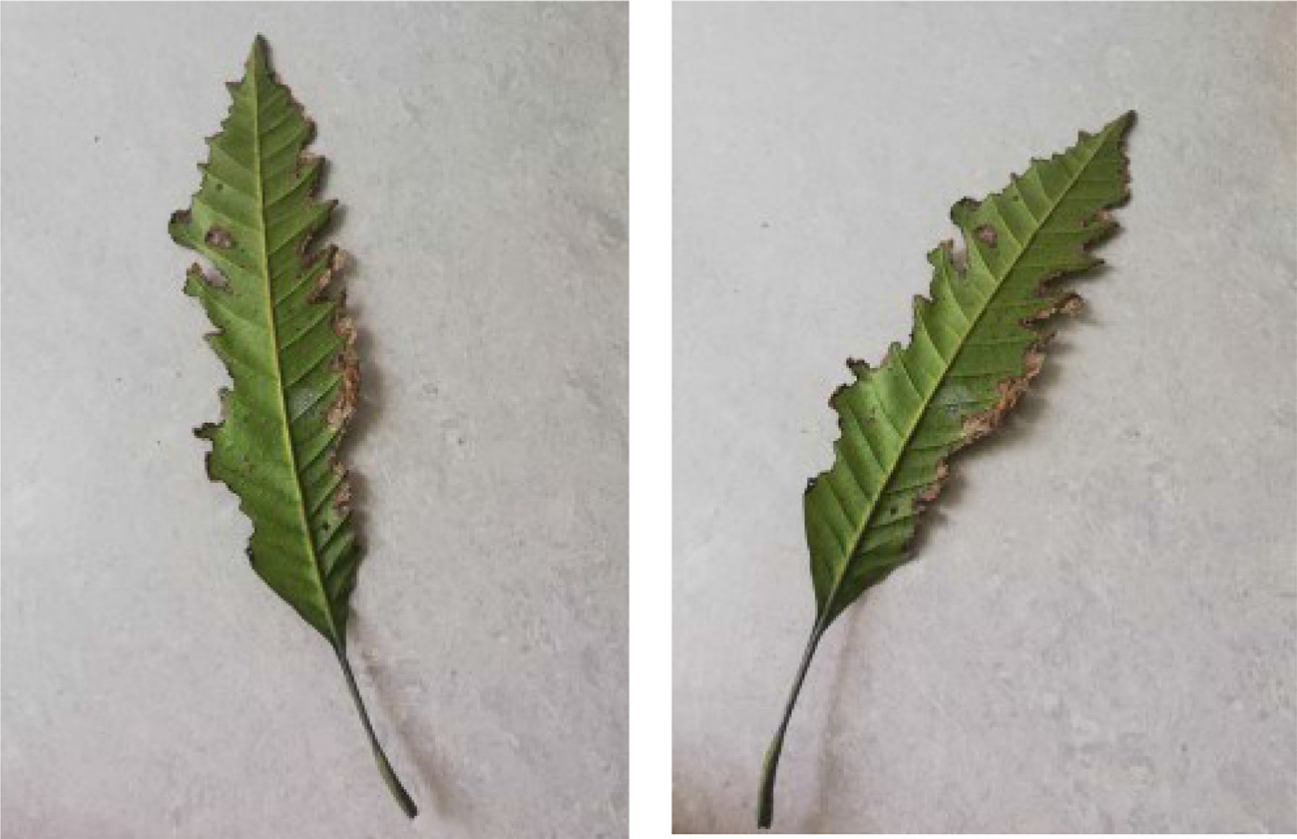
Bacterial canker.

**Fig. 3:**
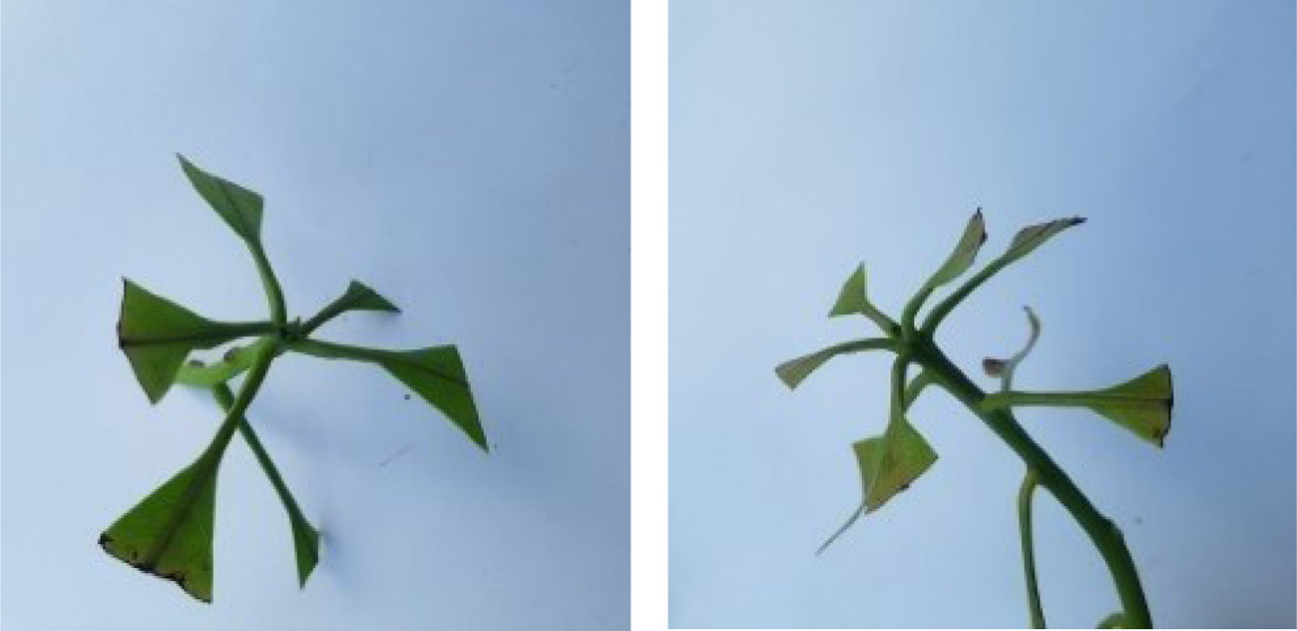
Cutting weevil.

**Fig. 4:**
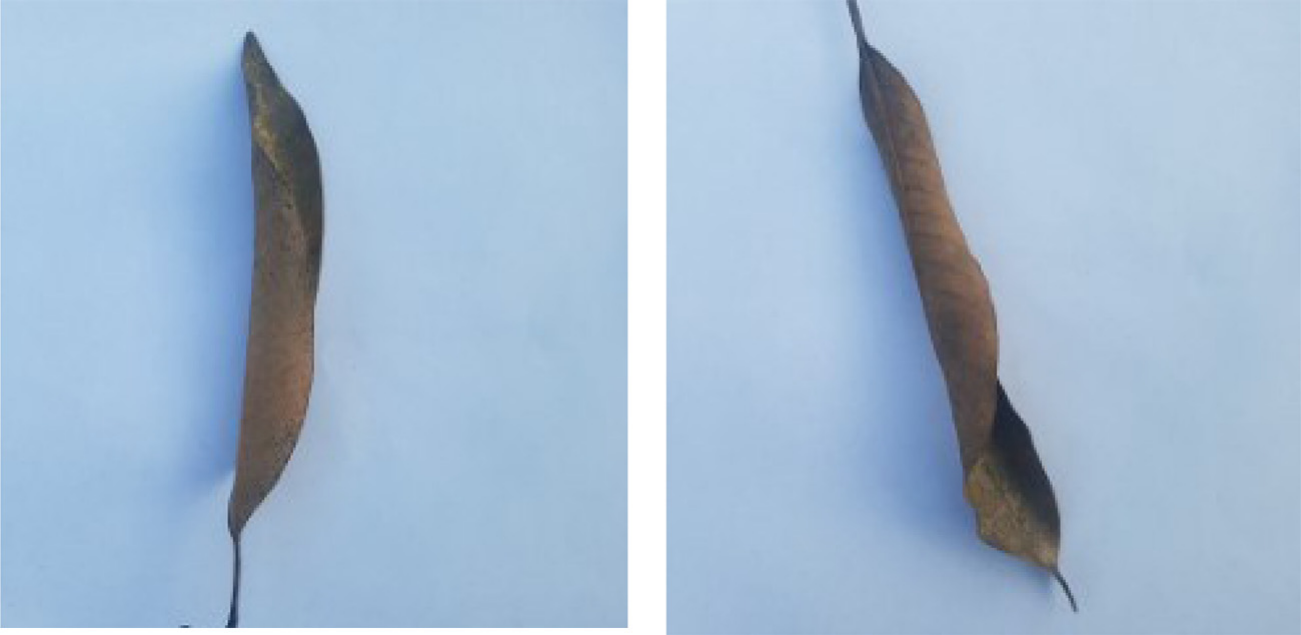
Die back.

**Fig. 5:**
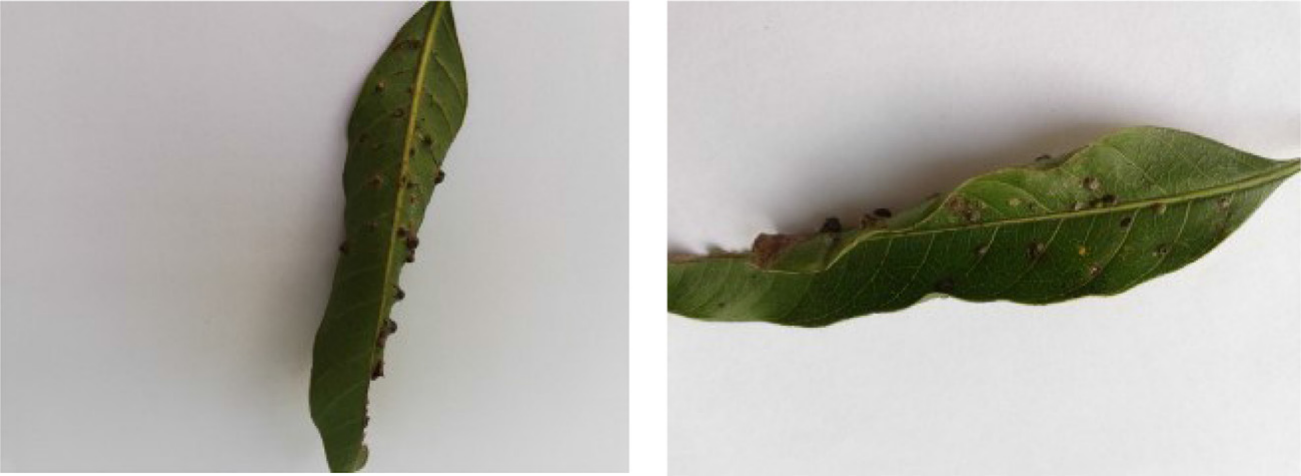
Gall midge.

**Fig. 6:**
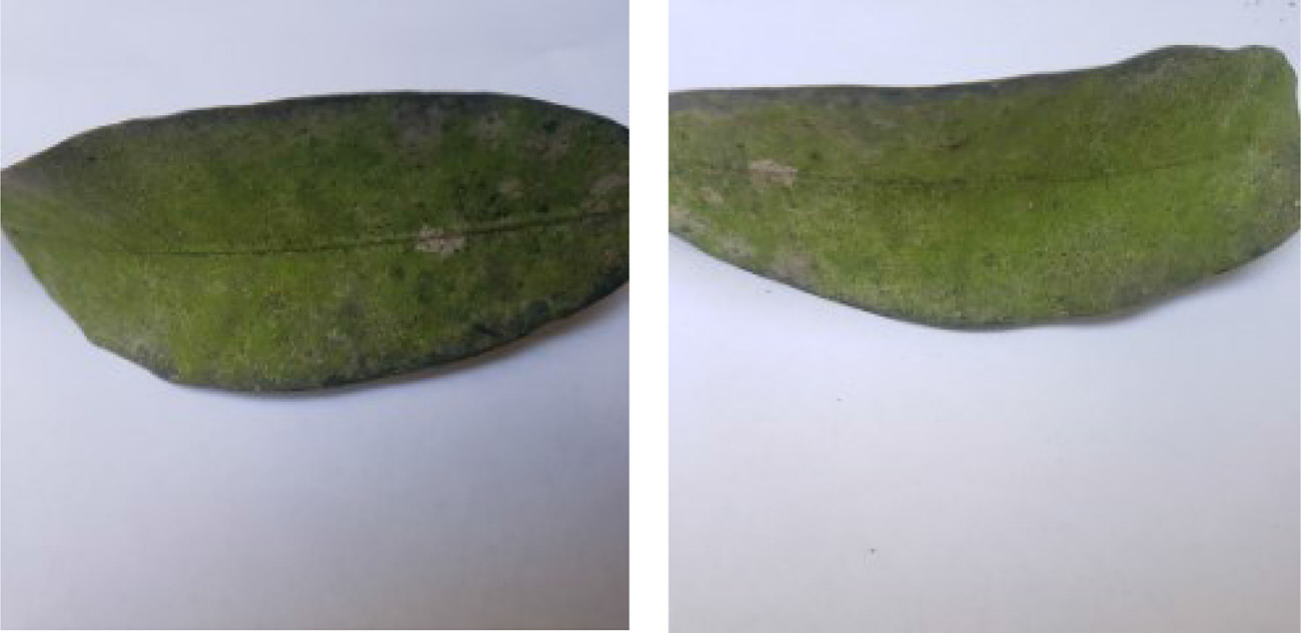
Powdery mildew.

**Fig. 7:**
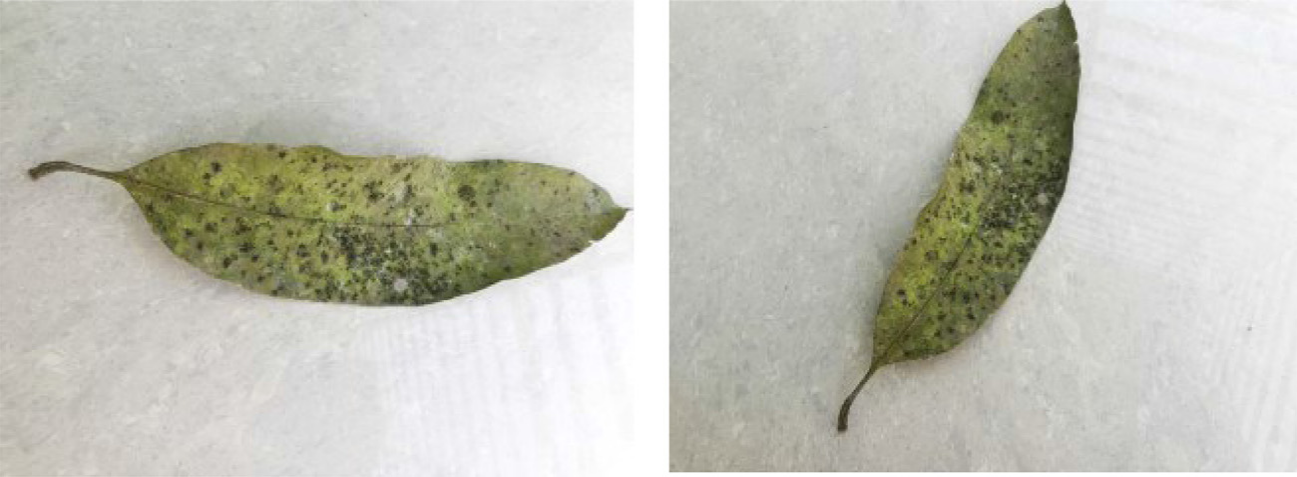
Sooty mold.

**Fig. 8:**
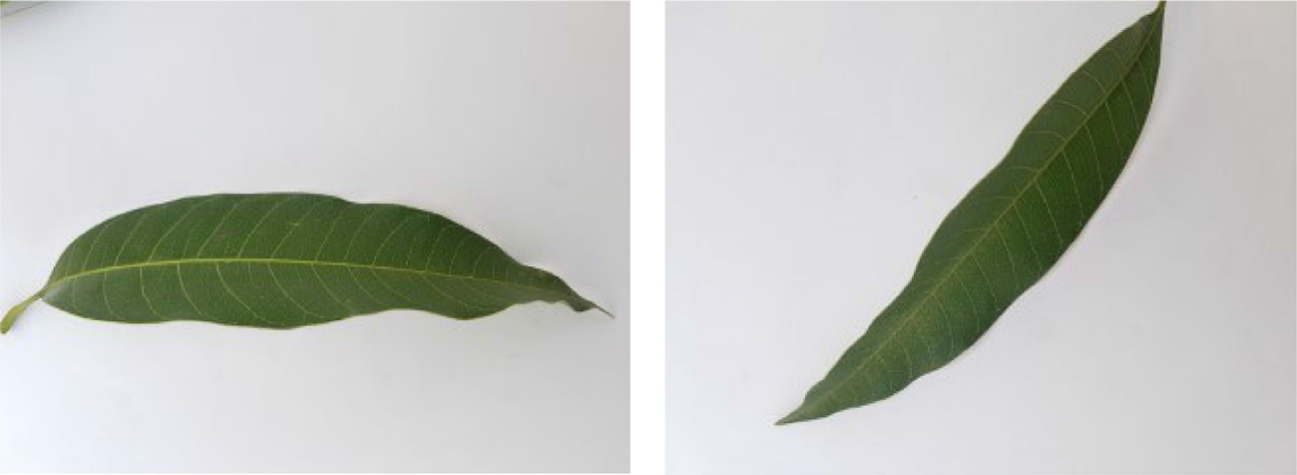
Healthy.

If we want to predict the labels of instances of a dataset using a machine learning model, the instances belonging to different classes pertaining to the dataset must have distinctive traits so that the model can effectively distinguish among the inter-class feature vectors during the prediction phase. In this section, we analyze the distinct traits of various diseases found in the leaf images of our dataset. Fig. 1, Fig. 2, Fig. 3, Fig. 4, Fig. 5, Fig. 6, Fig. 7, Fig. 8 show two sample images of each of the seven diseases and of healthy category.

When Anthracnose is present, black necrotic patches emerge on both sides of the mango leaf. In most cases, necrotic patches form along the leaf margins where the lesions merge. Leaves that have been severely affected begin to curl. Young tissue is the primary site of infection, but conidia can be seen in lesions of all ages; see Fig. 1. When infected by Bacterial Canker, the bacterium pseudomonas mangifera causes mango fruits, leaves, stalks, and branches to get water-soaked spots that turn into typical cankers; see Fig. 2. Cutting Weevil disease cuts the mango leaf in such a way that it looks like it is cut with scissors; see Fig. 3. Dieback is a disease that causes mango twigs to dry out and break off from the top down. This is followed by the leaves turning brown, drying out, and falling off; see Fig. 4. Gall Midge disease causes leaves to have what looks like pimples on them. Heavy outbreaks of mango Gall Midge disease result in defoliation and reduced fruit yield; see Fig. 5. The white, powdery growths of fungus on the surface of leaves, flower stalks, flowers, and young fruits are a sign of a disease called Powery Mildew; see Fig. 6. Honeydew is a sticky, sweet secretion that some insects make to attract other insects. The sooty mould grows on honeydew. Using the honeydew as food, the mould slowly spreads over the surface of the affected plant part, turning it black in different ways; see Fig. 7.

So we can see from the above discussion that there are reasonably distinctive features among various disease classes of the images, thereby making it a fertile domain for applying machine learning models.

## 3 Experimental Design, Materials and Methods

While traditionally plant diseases are identified by farmers and crop experts by visually examining the plants, in recent times the rapid surge of technological innovations has paved the way to use technologies to assist the humans in effective diagnosis of various plant diseases in relatively less time and cost. Among the cutting-edge technologies of today, the machine learning discipline stands high as these techniques can predict future events at an astonishing level, provided sufficient and appropriate past data are available to them. Harnessing the low-cost computer hardware, machine learning algorithms are being successfully applied in a range of human sectors including agriculture [6,10,16,19].

The key to the successful use of machine learning is having a good dataset to begin with. These algorithms extract hidden patterns from the dataset (which is called learning or training the model) and then, based on these learnt patterns, they predict future events. So there is a high correlation between the quality of the dataset and the performance of a machine learning system [[21], [22], [23]]. The quality of a dataset can be weighed in terms of its size, intra-class integrity, inter-class dissimilarity, degree of imbalance in data distribution across different classes, and absence of noise in the data and labels, among other aspects. Having these qualities makes a dataset standard. A dataset must be a true representative of the real-life scenario where the learning system is going to be applied; otherwise, the power of these models cannot be utilized^1^ [24]. Hence, different datasets are needed for different prediction tasks, and this makes the datasets, in and of itself, valuable. However, real-life dataset for plant disease detection is not plentiful in the research arena. In this research, we aim to develop a standard, ready-to-use, and publicly available dataset of images of mango leaves of Bangladesh. Mango is one of the most popular fruits in the world (specifically, the 5th or 6th most popular food in terms of production with a yearly yield of about 50 million metric tons [1,2]). However, few counties are eligible for growing disease-free mangoes^2^. Although Bangladesh is the 9th highest mango-producing country in the world [3], there is, to the best of our knowledge, no publicly available dataset consisting of mango leaves of Bangladesh containing ground-truth labels of leaves infected by Anthracnose, Bacterial canker, Cutting Weevil, Die Back, Gall Midge, Powdery Mildew and Sooty Mould.

## 4 Methodology

The role of data is tremendously important in machine learning to the extent that it is believed by the practitioners that it is the quality and quantity of the data, and not the mathematical model, that mostly influences the performance of modern machine learning systems. In technical terms, this philosophy is called the data-centric, as opposed to model-centric, approach [4]. That is why researchers must follow a set of best practices from the beginning to the end of the dataset preparation procedure. These steps include, but are not limited to: selecting representative samples from the real-world, cleaning the data, augmenting the data, and labeling the data. In this section, we describe the steps we take in our dataset preparation task.

Steps of dataset collection and preparation: The main phases of our entire dataset preparation procedure are as follows:

(1) Acquiring background knowledge on prevalent diseases that affect the mango trees.
(2) Selecting the mango orchards for data collection in consultation with agricultural experts.
(3) Physically capturing the images of healthy and diseased mango leaves from the trees. As mentioned earlier, we consider seven diseases in total.
(4) Validating the images of the dataset. This step includes: manually labeling the images by human experts, resizing the images to standard shape as practiced by machine learning researchers, and cleaning the images from background noise.

Below we elaborate each of the above-mentioned four steps:

*Studying the common diseases of mango leaves.* Various diseases of mango trees greatly affect the yield. Many of these diseases are manifested in the leaves of a tree. Common such diseases include Dieback, Powdery Mildew, Red Rust, Cutting Weevil, Bacterial Canker, Sooty Mould, Anthracnose, Gall Midges, etc. Studies [9] show that about 39% of mango trees are affected by Anthracnose whereas Powdery Mildew damages up to 23% of unsprayed trees. Bacterial Canker is a deadly disease that can damage mango yields by 10% up to 100% [9]. These considerations give us a glimpse of the danger of not detecting diseases at an early stage. Next, we discuss the main diseases that affect mango trees and exhibit visually noticeable foliar symptoms. Anthracnose is produced by the fungus Colletotrichum gloeosporioides and it is considered to be the most devastating disease [5]. Bacterial Canker is another deadly mango disease caused by *Xanthomonas axonopodispv* [7,17]. The mango leaf Cutting Weevil is a destructive insect that attacks newly emerging mango foliage [13]. Another significant disease is known as Die Back which is caused by the fungus *Lasiodiplodia theobromae* [8,15]. The larvae of a very small fly called *gall midge* feed within the plant tissue, causing some bulges on the leaves. These galls can cause damage to the mango plant's leaves, flowers, fruit, and shoots [12]. Powdery Mildew disease is caused by the fungus Oidium mangiferae, which is a plant pathogen that infects mango plants [11]. Sooty Mould, also known as Meliola Mangiferae, is one of the fungi that thrive on honeydew produced by sap-feeding insects. This fungus blocks sunlight from entering into the chloroplasts in the leaf, thereby interfering with the process of photosynthesis and damaging plant's growth [14].

*Selecting the mango orchards.* For data collection, four mango gardens have been selected in different parts of Bangladesh based on their sizes and varieties of trees. The selected orchards are: Sher-e-Bangla Agricultural University mango garden in Dhaka city, Jahangir Nagar University garden in Savar area, Udaypur village mango garden in Rajbari district, and Itakhola village mango garden in Nilphamari district. We select different locations of Bangladesh to minimize sampling bias. Our choice has been proved to be correct as we found a good amount ofleaves pertaining to various diseases from these gardens.

*Physically capturing the images.* In addition to the seven diseases mentioned earlier, since the machine learning models need to recognize healthy leaf images as well as disease-affected ones, we include the healthy images as the 8th category in our dataset. The leaf images were taken a few days before the winter of 2021. At first, the trees affected by the disease were located – oftentimes with the help of agriculture experts. Then, the affected leaf images were taken out from the trees. Finally, some healthy leaves were also taken from the trees. Some diseases were found in almost every tree whereas some other diseases were hard to find; for example, Bacterial Canker and Gall Midge disease-affected leaves were relatively low in number, which is natural. After collecting the leaves, the images of all the leaves were captured individually using a mobile phone camera by placing a leaf on top of a white table. The technical specification of the camera was: 12-megapixel (f/1.7, 1.4-micron), and the device was Samsung Galaxy s7 Edge. There was an insignificant time delay between picking a leaf and capturing the photo of that leaf. Also, we captured the images in the respective mango orchard, so the temperature and humidity were the same as that of the trees. To mention the lighting condition, temperature, humidity etc., we took the images on a typical day of pre-winter season where the weather was neither cloudy, nor foggy. This way a total of around 1800 images are taken where eight categories of leaves are present.

*Validating the images of the dataset.* Since the image size of machine learning models must be of the same size, each of the captured images is resized to 240 × 320 pixels for better visualization, and is saved in PNG format. The noises such as scratches present in the images are manually cleaned, and in this process, some severely hazy and noisy images are dropped out. This way a total of around 1800 images are recorded. After that, in order to facilitate variations in the images, zooming and rotation are performed on some images that result in 4000 images in total, where each of the eight categories has exactly 500 images. Note that there were a few leaves where traits of multiple diseases were present; we omit such leaves to reduce the noise in the dataset. (To be able to successfully classify unseen instances using a machine learning algorithm, there must be distinguishable patterns among various categories of the dataset.) Although we ourselves did study thoroughly the traits of leaves affected by different diseases, sometimes we corroborated our judgement on labeling the images with agricultural experts. This makes the quality of labeling even more reliable.

**Fig. 9:**
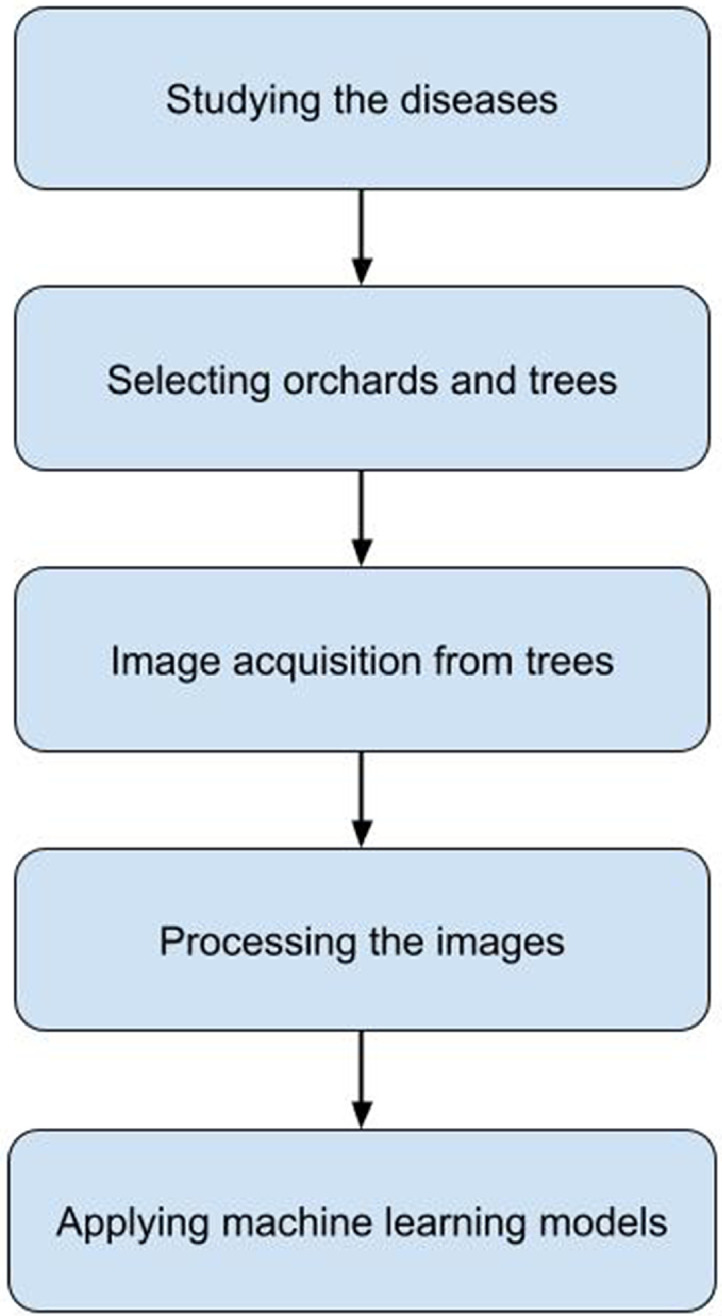
Flowchart showing the data preparation steps.

The flowchart of data preparation stages is shown in Fig. 9. Table 1 depicts all the information of the dataset at a glance.

**Table 1:** MangoLeafBD dataset information at a glance.

Type of data:	240 × 320 mango leaf images.
Data format	JPG.
Number of images	4000 images. Of these, around 1800 are of distinct leaves, and the rest are prepared by zooming and rotating where deemed necessary.
Diseases considered	Seven diseases, namely Anthracnose, Bacterial Canker, Cutting Weevil, Die Back, Gall Midge, Powdery Mildew, and Sooty Mold.
Number of classes	Eight (including the healthy category).
Distribution of instances	Each of the eight categories contains 500 images.
How data are acquired	Captured from mango trees through the mobile phone camera.
Data source locations	Four mango orchards of Bangladesh, namely, Sher-e-Bangla Agricultural University orchard, Jahangir Nagar University orchard, Udaypur village mango orchard,and Itakhola village mango orchard.
Where applicable	Suitable for distinguishing healthy and diseases leaves (two-class prediction) as well as for differentiating among various diseases (multi-class prediction).

## 5 Challenges to Prepare a Leaf Image Dataset

Preparing a machine learning dataset from scratch is not a trivial task; rather it is tedious. It requires significant human resources and time. But eventually, this effort, time, and labor pay off since a well-prepared dataset, if released for public use, is utilized by thousands of machine learning practitioners and researchers. Below we list the key challenges faced during our dataset preparation task:

• Diversity of leaf diseases: there is a large number of mango diseases.
• Geographical spread of mango orchards: researchers need to carefully choose the orchards and trees so that a good number of leaves of various diseases are taken into account.
• Technical difficulties during image acquisition: mango trees are quite tall so it is difficult to get physically close to the leaves.
• Many choices of data validation techniques: it requires a decent background knowledge on data science and machine learning to decide which methods will be useful and which are not.

## Ethics Statement

Not applicable.

## CRediT authorship contribution statement

**Sarder Iftekhar Ahmed:** Conceptualization, Methodology, Software, Investigation, Data curation, Visualization, Writing – original draft, Writing – review & editing. **Muhammad Ibrahim:** Conceptualization, Methodology, Visualization, Supervision, Validation, Writing – original draft, Writing – review & editing. **Md. Nadim:** Software, Investigation, Data curation. **Md. Mizanur Rahman:** Investigation, Data curation. **Maria Mehjabin Shejunti:** Investigation, Data curation. **Taskeed Jabid:** Methodology, Writing – review & editing, Supervision. **Md. Sawkat Ali:** Conceptualization, Methodology, Supervision, Visualization, Project administration, Validation.

## Declaration of Competing Interest

The authors declare that they have no known competing financial interests or personal relationships that could have appeared to influence the work reported in this paper.

## Data Availability

MangoLeafBD Dataset (Original data) (Mendeley Data)

## Footnotes

1 In technical terms, the distribution of the training set and test set should be similar. This is one of the major foundations of machine learning.

2 The top 10 mango producing countries produce almost 40 out of approximately 50 million metric ton yearly yield [3].
